# Chronic obstructive Eustachian tube dysfunction: CT assessment with Valsalva maneuver and ETS-7 score

**DOI:** 10.1371/journal.pone.0247708

**Published:** 2021-03-02

**Authors:** Diletta Angeletti, Annalisa Pace, Giannicola Iannella, Valeria Rossetti, Andrea Colizza, Irene Claudia Visconti, Giampiero Gulotta, Daniela Messineo, Marco de Vincentiis, Antonio Greco, Ferdinando D’Ambrosio, Giuseppe Magliulo

**Affiliations:** Department of “Organi di Senso”, Sapienza University of Rome, Rome, Italy; University of California, Davis, UNITED STATES

## Abstract

Chronic obstructive Eustachian tube dysfunction (ETD) is a common disorder of the middle ear. In recent years, two main diagnostic tools have become available: Eustachian tube score (ETS-7) and computed tomography (CT) combined with Valsalva maneuver. The aim of this study is to evaluate the outcomes of ETS-7 and CT in a group of patients affected by middle ear atelectasis with a strong suspicion of ETD. Three males and nine females, affected by middle ear atelectasis with retraction of the TM were enrolled. Each patient underwent to Eustachian tube dysfunction evaluation adopting the ETS-7 score and a temporal bone CT with Valsalva maneuver. The ears analyzed at steady state were divided into 2 groups: ETS<7 group and ETS≥ 7 group. The same division was applied for the ears analyzed after the Valsalva maneuver: ETS<7 group and ETS≥ 7 group. ETs were categorized as “well defined” (WD) and “not defined” (ND). The results of the analysis of the ETS-7 score in all 24 ears showed that 42% presented ETS ≥7, while 58% had ETS <7, indicating a diagnosis of ETD. In the ETS<7 group after Valsalva, ET was visualized in 33% of patients. In the ETS≥7 group it was WD in 29% after the Valsalva manoeuver. In both groups the comparison between the visualization of the ET before and after the Valsalva manoeuver did not present a statistical difference. No correlation emerged between ET evaluation with CT scan during Valsalva maneuver and ETS-7 score. It confirms that there is not a gold standard for the study of ET dysfunction.

## Introduction

The Eustachian tube (ET) extends anatomically from the middle ear to the nasopharynx and has an average length ranging between 31 and 38 mm in adulthood. It consists of a posterolateral bony portion, mainly formed by the petrous part of the temporal bone, and a fibrocartilaginous anteromedial portion, which provides structural support and allows mobility. At rest, the lumen of the Eustachian tube is closed: it opens temporarily during activities such as swallowing or yawning or with mandibular movement involved in important functions such as protection, clearance, middle ear ventilation and balancing of pressure changes between the middle ear and the environmental atmosphere [1].

Chronic obstructive Eustachian tube dysfunction (ETD) [2] is a common and uncomfortable disorder that persists for more than three months. It may compromise middle ear clearance and ventilation and be a potential cause of otitis media, tympanic membrane (TM) retraction pockets and cholesteatoma [3]. Due to the remarkable interest regarding the diagnosis and management of the ET that has never waned, in recent years two main diagnostic tools have been proposed for evaluating Eustachian tube function: Eustachian tube score (ETS-7) [4–7] and computed tomography (CT) combined with Valsalva maneuver [8–11].

ETS-7 is a relatively simple method for assessment of ET that provides reliable information about its active and passive dysfunction. Obstructive ETD (OETD) and Patulous ETD (PETD) are researched with signs and symptoms evoked by two maneuvers: Valvalva maneuver, that studies passive activity (failure of pressure related-opening), and Toynbee maneuver that tests active function (failure of muscle controlled opening). In particular, OETD may have a predominant active or passive dysfunction [12].

The difficulty in analyzing ET derives from the fact that there is no reliable test for determining ET function, meaning that diagnosis is based on clinical history alone.

ETS-7 has been used for evaluating ET opening in adult patients with OME and it has become increasingly popular, particularly for routine assessment before and after ET balloon dilation.

Imaging could be considered a non-invasive solution for assessing ET function, especially in surgical skull base surgery planning or tuboplasty.

Tarabichi et al. [9] first evaluated the feasibility of using the CT scan with the Valsalva maneuver in order to visualize the cartilaginous Eustachian tube lumen. The Valsalva maneuver allowed visualization of the whole length of the tube in 27/76 (35%) ears examined. It consistently visualized the distal third of the cartilaginous tube in 71/76 (94%) ears. Paradoxical collapse of the Eustachian tube was present in three ears. Other authors subsequently confirmed these findings, reporting how this technique might be helpful in localizing Eustachian tube pathology in patients with obstructive tube symptoms [8,11].

To our knowledge, in the literature no investigations have been described in which these two methods were correlated in any pathological entity potentially linked with ETD.

This study was designed in an attempt to evaluate their outcomes in a group of patients affected by middle ear atelectasis with a strong suspicion of ETD.

## Materials and methods

This research study was performed in accordance with the principles of the Declaration of Helsinki and approved by Sapienza Institutional Review Board (IRB) RIF.CE 4842 20–06.2019. All patients gave written informed consent for the tests to evaluate Eustachian tube function and CT scan.

Twelve patients, 3 males and 9 females, affected by middle ear atelectasis with retraction of the TM were enrolled in our prospective clinical study between January 2015 and September 2019. The patients’ ages ranged from 13 to 71 years, with a mean of 42 years.

Exclusion criteria included: no previous ear surgery, presence of rhino-pharynx neoformations and or hypertrophic adenoids.

All patients underwent a thorough ENT examination including micro-otoscopy and fiberoptic evaluation of the rhinopharynx. The ears were evaluated individually, and each ear was considered independently as a single data point. Middle ear atelectasis was classified using the Erasmus Atelectasis Classification system, proposed by Borgstein et al. [3]. This classification grouped middle ear atelectasis into 5 stages: Stage 1, tympanic membrane atrophic but not adherent; Stage 2, tympanic membrane adherent to the promontory; Stage 3, tympanic membrane adherent to incus or stapes; Stage 4, adherent to ossicles with retraction pocket but without cholesteatoma; Stage 5, retraction pocket with cholesteatoma or breakthrough. In order to render the study group as homogeneous as possible, we decided to select only patients presenting bilateral stage 3, ruling out all the other conditions. This justifies the small number of patients in the series and the relatively long time necessary for performing it.

### ETS-7

Eustachian tube dysfunction evaluation was performed adopting the ETS-7 score proposed by Schroder et al. [6] which comprises seven parameters able to assess the function of the ET: a) tubomanometric measurements (TMM) of ET function at three different pressure levels (30, 40 and 50 mbar); b) subjective estimations concerning the feasibility of Valsalva’s symptoms; c) subjective estimations of Toynbee’s clinical symptoms; d) tympanometry; e) objective Valsalva evaluation. The principle of TMM is the controlled delivery of defined pressures of 30, 40 and 50 mbar to the nasopharynx through a nasal applicator. The pressure receptor probe is positioned in the ear canal, and the nasal applicator is positioned in both nostrils. A pressure receptor probe located in the external ear canal registers pressure changes transmitted through movements of the TM/perforated TM. If the ET opens during swallowing, the defined pressure applied to the nasopharynx is transmitted into the middle ear spaces. The opening latency index (R value) reflects the latency between pressure application in the nasopharynx and the measurement of a pressure change in the ear canal. This latency quantifies Eustachian tube function: immediate opening (R<1) indicating good Eustachian tube function and late opening (R>1) indicating restricted Eustachian tube function. No opening (R negative or not measurable) indicates complete obstruction of the Eustachian tube. The parameters adopted for the calculation of the ETS-7 score are shown in Table 1.

**Table 1:** Parameters for the ETS-7 calculation.

Symptom/Finding	2 Points	1 Point	0 Points
Clicking sound when Swallowing	Always	Occasionally	Never
Positive subjective Valsalva	Always	Occasionally	Never
Objective Valsalva	Immediate	Immediate weak and slow	Negative
Tympanometry	A	B	C
TMM 30 mbar	R<1	R>1	No R
TMM 40 mbar	R<1	R>1	No R
TMM 50 mbar	R<1	R>1	No R

Regarding TMM results at 30, 40 and 50 mbar, an immediate opening of the ET (R<1) is given 2 points, a delayed opening (R>1) 1 point and no opening (negative or not measurable R) 0 points.

ETS-7 score ranges from a minimum of 0 to a maximum of 14 points and it was calculated for all the patients of the study group. An ETS-7 result < 7 corresponds to a diagnosis of chronic ETD.

### CT scan with Valsalva

A temporal bone CT examination covering the entire area of the ET and its surrounding tissues was performed. High-resolution temporal bone CT scans were performed with the patient in a supine position. Helical images were acquired without contrast and were reconstructed using a bone plus algorhythm. The slice thickness was 1 mm. All the patients underwent CT scan in steady state and during the Valsalva maneuver [9].

After obtaining the patient’s consent and indication to the investigation, a CT scan of the temporal bone was performed first in basal conditions and then with the patient actively performing the Valsalva maneuver. The technician instructed the patient that the second acquisition would take place during a strong exhalation with the nose closed. The primary image acquisition was performed in a supine position. The acquisition was performed in volumetric mode. Images were acquired using Toshiba Aquilion16 channel CT equipment and MPR Software for multiplanar reconstructions "in real time". This equipment uses high efficiency solid state detectors with a high number of detectors and high sampling rate. Acquisition parameters were: high contrast spatial resolution (lp/cm) the best possible (at 50% of the MTF curve). The scan parameters used were: mA 100/ 0.5 mAs, KV 120, 1 mm layer, with FOV 50 cm. overlap, 0.67 mm (thickness) 0.5 mm (increment). Multiplanar reconstruction of the images on the Eustachian channel axis was performed using proprietary Toshiba software. The following methodology was applied for image processing: the centre of rotation was positioned on the bottom of the nasopharyngeal end of the Eustachian tube on axial sections. Using the scouts, the axial plane was then tilted downwards and forwards until the entire length of the Valsalva canal was displayed.

### Statistical analysis

ETS-7 was calculated for all ears studied, and was estimated the percentage of patients (x/24) with ETS-7<7 and ETS-7≥7. It was estimate the percentage of patients with a “well defined” (WD) Eustachian tube on CT and the percentage of “not defined” (ND) Eustachian tube considering all the sample (WD/24 and ND/24) at steady state and after Valsalva maneuver.

There was performed the comparison of visualization of ET (WD and ND) before and after Valsalva maneuver, in both ETS-7 groups, with Fischer’s exact test. A p value of <0.05 was considered statistically significant.

It was calculated the R values of each single ear at three pressures (30, 40, 50 mbar) and was estimated the percentage ear with each value (ex: n°R values<1 at 30mbar/24).

There was performed the comparison between the number of ears with R<1 and R>1 by Fischer’s exact test. A p value of <0.05 was considered statistically significant.

## Results

The results of the ETS-7 score, showed that 10/24 (42%) ears have a score ≥7, while in 14/24 (58%) ETS-7 was <7, indicating a diagnosis of ETD.

The ET anatomy was evaluated with CT scan at steady state and during the Valsalva maneuver. ETs were categorized as “well defined” (WD) when CT clearly visualized the whole length of the Eustachian tube, on the contrary non visible ETs were considered as “not defined” (ND). (Table 2)

**Table 2:** Results.

	Age-Sex	Atelectasis Stage	ETS-7 score	TMM 30 mbar	TMM 40 mbar	TMM 50 mbar	TC pre Valsalva	TC post Valsalva
Pt 1 (R)	58y F	3	1	0	0	0	WD	WD
Pt 1 (L)	58y F	3	2	0	0	0	WD	WD
Pt 2 (R)	22y M	3	7	1	1	2	ND	WD
Pt 2 (L)	22y M	3	7	1	1	2	ND	WD
Pt 3 (R)	15y F	3	5	1	1	2	ND	ND
Pt 3 (L)	15y F	3	8	1	2	2	ND	ND
Pt 4 (R)	71y F	3	3	0	1	1	WD	WD
Pt 4 (L)	71y F	3	1	0	0	0	WD	WD
Pt 5 (R)	56y F	3	6	1	2	1	WD	WD
Pt 5 (L)	56y F	3	7	1	2	2	WD	WD
Pt 6 (R)	24y F	3	6	1	1	2	ND	WD
Pt 6 (L)	24y F	3	1	0	0	1	ND	WD
Pt 7 (R)	47y F	3	7	0	0	0	WD	WD
Pt 7 (L)	47y F	3	8	2	2	2	WD	WD
Pt 8 (R)	34y F	3	8	2	2	2	ND	ND
Pt 8 (L)	34y F	3	6	0	2	2	ND	ND
Pt 9 (R)	61yM	3	7	1	2	2	ND	ND
Pt 9 (L)	61y M	3	1	0	0	0	ND	ND
Pt 10 (R)	56y F	3	1	0	0	0	ND	ND
Pt 10 (L)	56y F	3	1	0	0	0	ND	ND
Pt 11 (R)	13y M	3	1	0	0	0	ND	WD
Pt 11(L)	13y M	3	1	0	0	0	ND	ND
Pt 12 (R)	49y F	3	7	1	2	2	WD	WD
Pt 12 (L)	49y F	3	7	1	2	2	WD	WD

Pt = patient; Atelectasis stage classified by the Erasmus Atelectasis Classification; WD = well defined; ND = not defined; F = female; M = male; CT = computerized tomography; (R) = right ear; (L) = left ear.

At resting state, 10/24 (42%) ETs were “well defined” (WD) while the other 14/24 (58%) were “not defined” (ND).

The ears analyzed at steady state were divided into 2 groups: ETS<7 group and ETS≥ 7 group. The same division was applied for the ears analyzed after the Valsalva maneuver: ETS<7 group and ETS≥ 7 group.

**Fig 1:**
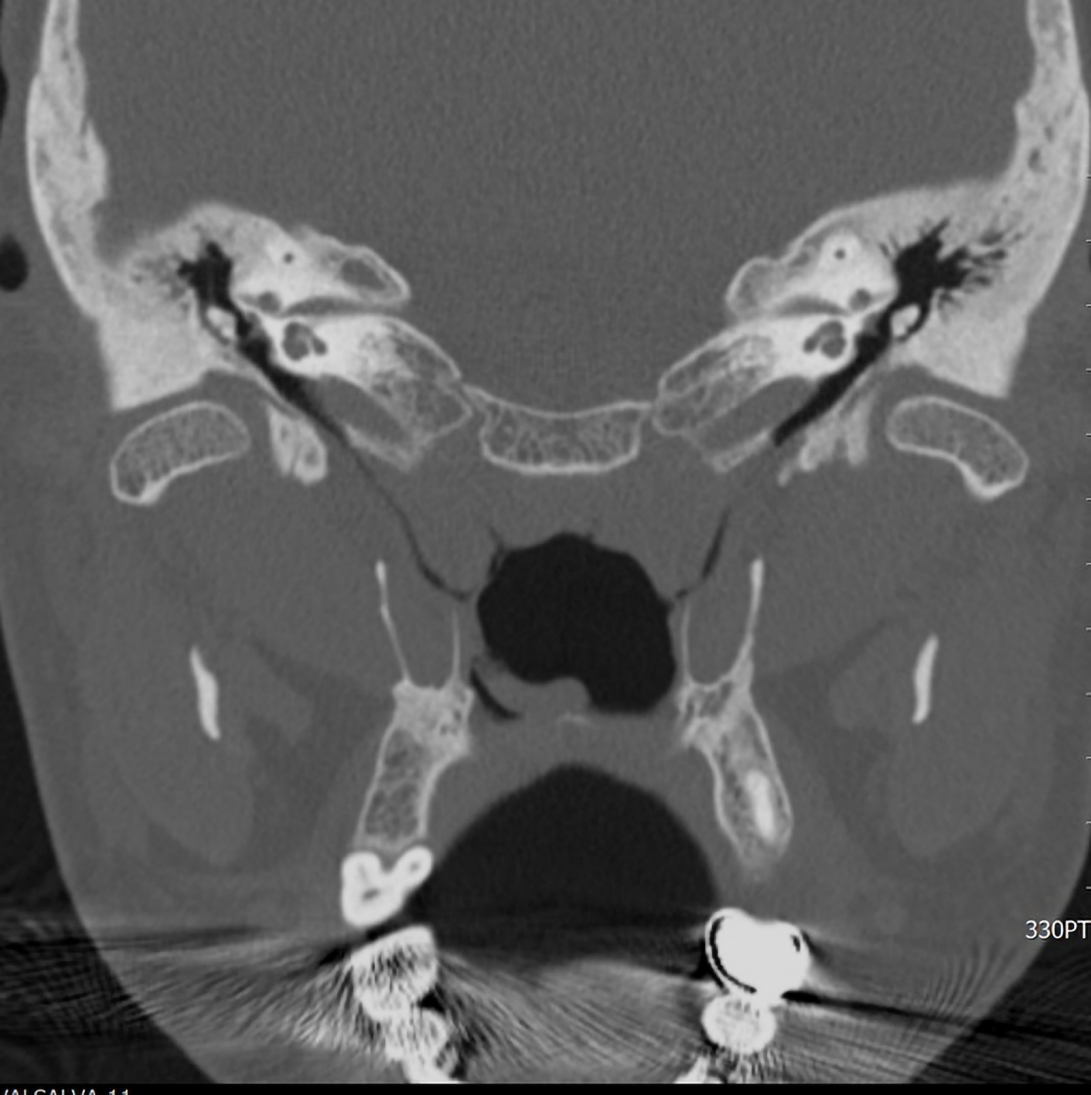
Eustachian tube visible after Valsalva maneuver.

**Fig 2:**
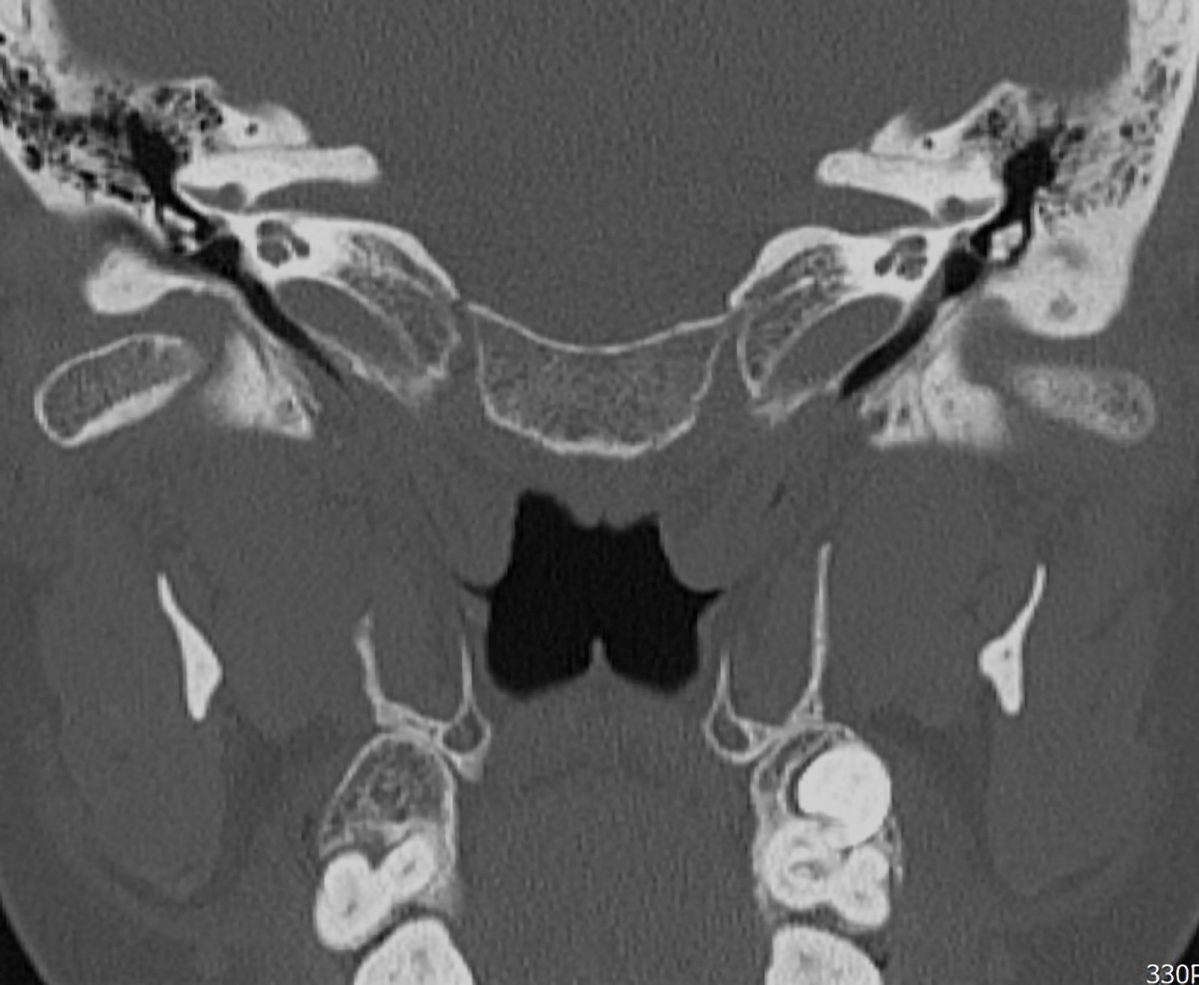
Eustachian tube not visible after Valsalva maneuver.

**Fig 3:**
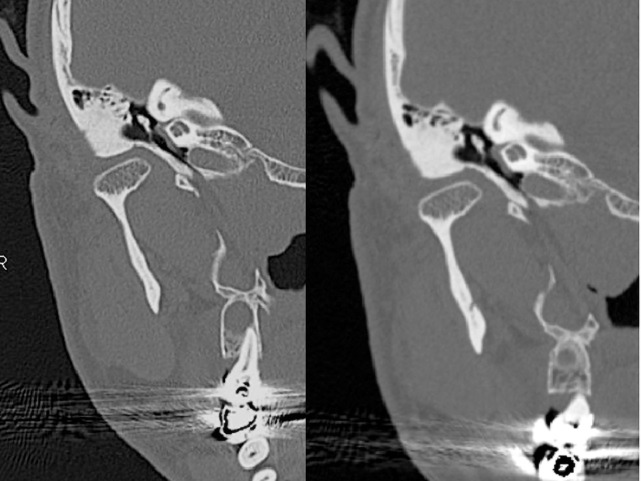
Eustachian tube only minimally visible before and after Valsalva maneuver.

At steady state, CT scan showed that in ETS<7 group 5/14 cases (36%) have a WD ET, while 9/14 cases (64%) presented a ND ET. CT scan during the Valsalva maneuver in the ETS<7 group evidenced a WD ET in 8/14 (57%) cases and a ND ET in 6/14 (43%) (Figs 1–3).

In the ETS≥7 group CT scan at steady state showed 5/10 cases (50%) with a WD Eustachian Tube, while 5/10 cases (50%) had a ND Eustachian Tube. CT scan during the Valsalva maneuver showed 7/10 cases (70%) with a WD ET and 3/10 (30%) with a ND ET (Figs 1–3).

In ETS<7 group 2 cases passed from ND to WD after the Valsalva maneuver; in the ETS≥7 group 3 cases changed from ND to WD after the Valsalva maneuver (Table 3).

**Table 3:** Comparison between the two diagnostic tools in positive and negative ETD.

	ETS ≥7		ETS <7	
	SS	Valsalva	SS	Valsalva
**WD**	**5/10 (50%)**	**7/10 (70%)**	**5/14 (36%)**	**8/14 (57%)**
**ND**	**5/10 (50%)**	**3/10 (30%)**	**9/14 (64%)**	**6/14 (43%)**

ETS = Eustachian tube score; WD = well Defined; ND: not defined; SS = steady state.

Comparison between the visualization of the ET before and after the Valsalva maneuver in both ETS≥7 groups, did not show a statistical difference (p<0,6). The same was true when comparing ET before and after the Valsalva maneuver in both groups with ETS<7 (p<0,4).

The comparison between the change of ET status (WD vs ND), estimated in all ET, showed no statistical difference before and after the Valsalva maneuver (p<0,2).

All the percentages are summed up in Table 3.

As already mentioned, the ETS-7 score involves objective and subjective parameters. In an attempt to correlate the objective and dynamic parameters of TMM with those of the Valsalva maneuver during CT, we also calculated the R values of each single ear at the three pressures of 30, 40, and 50 mbar in order to rule out any eventual influence of the personal sensation of patients.

It was found that at 30 mbar only 2/24 (8%) ears scored 2 points (R<1), 10/24 ears (42%) 1 point (R> = 1) and **12/24** ears (50%) 0 points (negative or not measurable R); at 40 mbar 9/24 (37,5%) ears scored 2 points, 5/24 (21%) ears 1 point and 10/24 (41,5%) 0 points; at 50 mbar 12/24 (50%) ears scored 2 points, 3/24 (12,5%) ears 1 point and 9/24 (37,5%) 0 points (Table 3).

In accordance with the definition of normal function of ET with R<1, the number of ears that presented R<1 was compared to those with R>1: there was a statistical difference between the two values at 30 mbar vs 40mbar (p<0,03). Similar findings emerged comparing 30 mbar and 50 mbar (p<0.003). On the contrary, there was no statistical difference comparing the number of ears with R<1 at 40 vs 50mbar (p<0,56).

## Discussion

The clinical evaluation of ETD remains a source of controversy [12]. A potential diagnostic method for ETD classification was suggested in the research reported by Schroder et al [6]. In fact, these authors modified a score system, previously devised by Ockermann et al. [5], for assessing the functionality of the Eustachian tube. This classification, named ETS-7, combined a complex of subjective and objective parameters. It has certainly improved the diagnostic capabilities of ETD, but does not represent a definitive solution to the problem.

Nowadays, there is no reliable radiological test for determining ET function. The advent of high-resolution CT with multi-planar reconstruction led to a proliferation of ET imaging studies over the last decade with the aim of analyzing ET length and direction, in order to evaluate the 3D anatomy and surrounding tissues [8–11].

The morphological studies analyzed the anatomic structures of the Eustachian tube at rest. The introduction of the CT associated with the Valsalva maneuver makes it possible to follow the entire course of the ET from the rhino-pharyngeal orifice up to the bony orifice into the middle ear providing useful information regarding its ventilation. However, this imaging technique proved to be particularly expensive in terms of money and time.

A reliable test, useful for the evaluation of function and the detailed anatomical imaging of ET, could be helpful in follow up and for surgical planning.

Tarabichi et al, investigated the ability of the Valsalva maneuver to increase the visualization of the lumen of the cartilaginous tube in patients who did not present ear complaints, ear pathology or radiographic evidence of sinus disease [9].

The Valsalva maneuver showed a clear visualization on CT of the entire length of ET in 35% of the ears examined.

In accordance with the above findings, the aim of this study was to evaluate the possibility of ameliorating these values combining the use of the CT with the Valsalva maneuver and the ETS-7 score.

The first target was to select the patients in whom this diagnostic protocol should be carried out. Patients with middle ear atelectasis were chosen for their highly probable ETD and the sample was rendered as homogeneous as possible. This explains the limited number of cases and the long time needed to form the study group.

The results of the analysis of the ETS-7 score in all 24 ears showed that 42% presented ETS ≥7, while 58% had ETS <7, indicating a diagnosis of ETD.

The results of CT were not in line with those of Tarabichi. In the ETS<7 group after Valsalva, ET was visualized in 57% of patients. In the ETS≥7 group it was WD in 70% after the Valsalva manoeuvre. However, in both groups the comparison between the visualization of the ET before and after the Valsalva manoeuvre did not present a statistical difference.

This could be attributable to the subjective component that has to be taken into consideration. First of all, during CT the position assumed by the patients might make it difficult to carry out the manoeuvre properly exerting the correct pressure.

Moreover, as mentioned above, the ETS-7 score includes both subjective and objective parameters.

How might this subjective parameter influence results? In order to understand the real function of ET using only objective parameters, the focus was pointed solely on the variation of results on the TMM at 30-40-50 mbar. ET function is considered normal with a R<1. Comparing the variation of R at 30 mbar vs 40mbar it emerged that the difference between the number of R<1 and R>1 was statistically different. The same was true when comparing the variation between 30 mbar and 50 mbar (p<0.003). On the contrary, there was no statistical difference when comparing the number of ears with R<1 at 40 vs 50mbar (p<0,56).

This phenomenon represents an additional point of discussion. Probably, the absence of changes on CT images is related to the conditions of the exam. It may be possible that patients, in the supine position and in a narrow place, find it difficult to accomplish a correct Valsalva maneuver, thus not consenting opening of the ET. It is known, in fact, that during TMM known pressure levels are applied informing us at what pressure the tuba opens. On the other hand, the pressure necessary for obtaining opening of the tube with the Valsalva maneuver during CT cannot be quantified since it is too variable and unpredictable, influenced as it is by the specific and personal characteristics of each individual patient. Moreover, further studies with larger sample are ongoing to analyze ETS scores versus the CT results without pre-defining two groups based on ETS < or ≥ 7.

## Conclusion

These findings could indicate that as the pressure increases at the TMM it approaches the pressure reached applying the Valsalva maneuver. This is a result which encourages us to increase the small sample examined and to evaluate ET anatomy and pathophysiology in patients with middle ear atelectasis with other grades of tympanic retraction and after tympanoplasty. On the other hand, it confirms that there is not a gold standard for the study of ET dysfunction, likely due to the complexity of the anatomy and pathophysiology of this organ and to the actual limitations of the available technologies.

## References

[1] Alper CM, Luntz M, Takahashi H et al. Panel 2: Eustachian Tube, Middle Ear, and Mastoid Anatomy, Physiology, Pathophysiology, and Pathogenesis. OTO open 2013; 156(4): S22–S40. 10.1177/0194599812472631 23536530

[2] Schilder AGM, Bhutta MF, Butler CC, et al. Eustachian Tube Dysfunction: Consensus Statement on Definition, Types, Clinical Presentation and Diagnosis. Clin Otolaryngol 2015; 40 (5): 407–11. 10.1111/coa.12475 26347263 PMC4600223

[3] Borgstein J, Gerritsma T V, Wieringa MH, et al. The Erasmus Atelectasis Classification: Proposal of a New Classification for Atelectasis of the Middle Ear in Children. Laryngoscope. 2007;60:1255–9. 10.1097/MLG.0b013e31805d0160 17603325

[4] Sudhoff H, Schröder S, Reineke U, et al. Therapy of Chronic Obstructive Eustachian Tube Dysfunction: Evolution of Applied Therapies. HNO 2013; 61(6),477–82. 10.1007/s00106-013-2691-6 23515595

[5] Ockermann T, Reineke U, Upile T, et al. Balloon Dilatation Eustachian Tuboplasty: A Clinical Study. Laryngoscope Jul 2010;120 (7), 1411–6. 10.1002/lary.20950 20564474

[6] Schröder S, Lehmann M, Sauzet O, et al. A Novel Diagnostic Tool for Chronic Obstructive Eustachian Tube Dysfunction—The Eustachian Tube Score. Laryngoscope Mar 2015;125 (3), 703–8. 10.1002/lary.24922 25215457

[7] Magliulo G, de Vincentiis M, Iannella G et al. Eustachian tube evaluation in patients with obstructive sleep apnea syndrome. Acta Otolaryngol 2017; 138 (2), 159–164. 10.1080/00016489.2017.1385846 28990834

[8] McDonald M, Hoffman M.R, Lindell R. et al. New insights into mechanism of Eustachian tube ventilation based on cine computed tomography images. Eur Arch Otorhinolaryngol 2013; 269(8):1901–7. 10.1007/s00405-011-1829-y PMC3679544 22120826

[9] Tarabichi M, Najmi M. Visualization of the Eustachian Tube Lumen With Valsalva Computed Tomography. Laryngoscope 2015;125(3):724–9. 10.1002/lary.24979 25376511

[10] Syed AZ, Hawkins A, Alluri LS, et al. Rare finding of Eustachian tube calcifications with cone-beam computed tomography. Imaging Sci Dent 2017; 275–80. 10.5624/isd.2017.47.4.275 29279828 PMC5738511

[11] Alper CM, Rath TJ, Teixeira MS, et al. A Novel Imaging Method for the Cartilaginous Eustachian Tube Lumen: Computerized Tomography During the Forced Response Test. Ann Otol Rhinol Laryngol 2018; 127(1): 13–20. 10.1177/0003489417740363 29099232 PMC6190804

[12] Smith ME, Takwoingi Y, Deeks j, Alper C, Bance ML, Bhutta MF et al. Eustachian tube dysfunction: A diagnostic accuracy study and proposed diagnostic pathway. PLoS One. 2018 8;13(11):e0206946 10.1371/journal.pone.0206946 30408100 PMC6224095

